# Development, implementation, and evaluation of an undergraduate family medicine program in the United Arab Emirates

**DOI:** 10.1186/s12909-024-05134-6

**Published:** 2024-03-20

**Authors:** Nabil Sulaiman, Sarra Shorbagi, Salman Yousuf Guraya

**Affiliations:** 1 Department of Family and Community Medicine and Behavioural Science, College of Medicine, University of Sharjah, Sharjah, United Arab Emirates; 2 Department of Clinical Science, College of Medicine, University of Sharjah, Sharjah, United Arab Emirates; 3 Baker/ IDI Heart and Diabetes Institute, 75 Commercial Road, 3004 Melbourne, VIC Australia

**Keywords:** Family medicine, Medical students, Medical curriculum, Kern’s model

## Abstract

**Background:**

Healthcare systems rely on well-trained family medicine physicians who can offer continuous quality services to their communities and beyond. The American Academy of Family Physicians and the World Organization of Family Doctors recommend that medical curricula should have adequately supervised education and training of the learners in family medicine during their preclinical and clinical placements. However, some medical schools don’t have a comprehensive family medicine program to prepare graduates who can meet the community needs. This work aims to report the essential steps for the development, implementation, and evaluation of the family medicine program at the College of Medicine at the University of Sharjah in United Arab Emirates.

**Methods:**

We used the Kern’s 6-step model to describe the development, implementation, and evaluation of the family medicine program. This includes problem identification, needs assessment, goals setting, educational strategies, implementation, and evaluation. During 2014–2022, we longitudinally collected essential information about the family medicine program from different stakeholders including the feedback of clinical coordinators, adjunct clinical faculty, and medical students at the end-of-clerkship. All responses were analysed to determine the effective implementation and evaluation of the family medicine program.

**Results:**

Over the course of 8 academic years, 804 medical students, 49 adjunct clinical faculty and three College of Medicine faculty participated in the evaluation of the family medicine program. The majority of respondents were satisfied with various aspects of the family medicine program, including the skills gained, the organisation of program, and the variety of clinical encounters. The medical students and adjunct clinical faculty suggested the inclusion of e-clinics, faculty development program, and the expansion of more clinical sites for the effectiveness of the family medicine program.

**Conclusions:**

We report a successful development, implementation, and evaluation of the family medicine program in United Arab Emirates with a positive and impactful learning experience. More attention should be paid towards a suitable representation of family medicine program in the medical curriculum with focused and targeted educational plans for medical students.

**Supplementary Information:**

The online version contains supplementary material available at 10.1186/s12909-024-05134-6.

## Background

In the last few decades, the role of family medicine (FM) has gained increasing recognition in delivering better-quality patient care, equity in health throughout the population, and more efficient use of resources than specialty-oriented systems [1–3]. The World Health Organization (WHO) recommends that all countries should direct their healthcare systems towards strengthening primary health care [4]. The value of primary care to people and the healthcare system is more significant when care is delivered by trained practitioners [1, 5]. As such, healthcare systems require well-trained FM physicians who offer continuous and comprehensive healthcare needed by individuals and families. To meet this need, each country must provide physicians with rigorous educational plans focusing on the health problems of the population served [1, 4]. However, the percentage of trained FM physicians is less than the overall number of specialists in other disciplines globally [6]. This shortage of FM physicians has become a concern in many nations, mainly because medical graduates from different countries have shown little interest in FM as a career choice [7, 8]. One of the reasons for less interest in FM program could be students’ experiences during early clinical clerkships which substantially impact their attitudes and interests [9].

A recent review of the literature indicates that undergraduate FM programs offer early exposure to patients and introduce students to communication skills, doctor-patient relationships, and clinical skills training [10]. In the clinical years, FM training provides an opportunity for more patient contact and more time spent at clinical training sites than hospitals [10, 11]. Several factors have been associated with an high likelihood of students choosing family medicine as a career path. Participation in a mandated FM program during medical curriculum has shown a correlation with a higher likelihood of choosing a FM specialty [12]. Early exposure to FM clerkships during undergraduate years has also been found to influence graduates’ future career choices towards FM [11, 13, 14]. Longer clerkships duration, high quality practice, diverse patients’ exposure, broad spectrum primary care practices, as well as supportive environment were linked to a greater number of students opting for FM as a career specialty [15].

There is variability in the length and scope of training in undergraduate FM program across different countries. The European Academy of Teachers in General Practice and Family Medicine (EURACT) surveyed 259/400 medical schools in 39 European countries to investigate the availability of FM programs in European schools. A total of 50 medical schools, mostly in Southern and Eastern Europe, reported either a lack of or only very brief FM programs [16]. In the Eastern Mediterranean Region (EMR), there is a scarcity of published literature about the development and implementation the FM program curriculum [6, 11, 17]. In this paper we use the Kern’s 6-step model to describe the development, implementation, and evaluation of an undergraduate FM program at the College of Medicine (CoM), University of Sharjah (UoS) in United Arab Emirates (UAE), which is a part of the EMR. We also report on the findings of the evaluation of the FM program using end of clerkship feedback from students and faculty.

## Methods

We conducted a longitudinal study to evaluate the development and implementation of our FM program at the CoM in UAE.

### Study setting

The Bachelor’s Program in Medicine and Surgery at the CoM, established in 2004, is a six-year program divided into three phases: Phase I, Foundation Year; Phase II, Pre-clerkship Phase, which includes years 1, 2, and 3; and Phase III, Clerkship Phase for years 4 and 5. During year four students rotate every ten weeks around four clerkships: medicine, surgery, obstetrics and gynaecology, and paediatrics. While in year 5, students rotate every 10 weeks between medicine, surgery, and FM/psychiatry clerkships (6 weeks FM and 4 weeks in Psychiatry). The first batch of the FM program was conducted in the academic year 2009–2010.

### Study participants

We conducted this study on undergraduate medical students studying FM program at the CoM at UoS and the faculty who delivered this program over 8 years from 2014 to 2022.

### Data collection

The FM department utilised various sources of information to evaluate the FM program including end- of clerkship feedback of students as well as adjunct clinical faculty (ACF) to reflect on and share their experiences, discuss challenges, and offer solutions. The students’ survey at the end of the clerkship was distributed to explore their experiences about the training sites, number and variety of clinical encounters, feedback on their performance, and their overall satisfaction with the training at each site. Students’ survey consisted of ten questions, of which eight were prepared on a 5-point Likert scale. Students’ responses to other two open-ended questions were reviewed and summarised. Similarly, we collected reflections from ACF by sending end of clerkship surveys to record their insights about the scientific content, delivery, and assessment of the FM program.

### Data analysis

The students’ responses were analysed using excel spread sheet to generate bar charts representing percentages for each of the questions. Through several deliberations between NS and SS, the reflections of ACF were reviewed, collected, and summarised as strengths and areas for improvement of the FM program. To reduce bias, a second round of review was performed by SG. All researchers agreed on the final content of the ACF reflections.

## The process of program design, development, and implementation

We adopted Kern’s 6-step approach [18] for curriculum development which involves defining the problem, needs assessment, developing goals and objectives, educational strategies, implementation process and the evaluation of the program. These steps provide a systematic framework for designing educational programs. In the forthcoming sections of the manuscript, we have detailed the Kern’s 6-step model of our FM program development, implementation, and evaluation.

### Problem identification and needs assessment

A review of the needs for the FM training program was based on the 2015 WHO report which highlighted the annual output of trained family physicians from 22 countries in the EMR, with an average of around 0.08 FM physicians per 10,000 population [6]. Considering the current annual production and population projections until 2030, the expected ratio (excluding those leaving family practice or retiring) is projected to increase to 0.42 FM physicians per 10,000 population if countries maintain the same trends in FM postgraduate training. This number is far from the recommendations of the World Organization of Family Doctors (WONCA) with three to six family physicians per 10,000 population [19]. Therefore, countries in the region need to enhance their annual production of FM physicians [6].

At the undergraduate level, establishing FM program in medical schools is crucial as it serves the foundation for the specialty. Unfortunately, many medical schools in the EMR lack exposure to FM in their undergraduate programs. To address this gap, there is a need to create FM department in all medical schools, allowing medical students to gain exposure and understanding of this specialty. Without proper training at the undergraduate level, students miss an important opportunity to gain experience in FM concepts. Transforming the system from a general practitioner-based to FM physician-based system requires early exposure and recognition of FM as a valuable career option. It’s important to note that medical education in most institutions in the EMR is predominantly hospital-based, but it should ideally involve a combination of hospital and community-based activities at the undergraduate level [6]. This decision aligns with the commitment of the CoM at the UoS to provide a practical educational experience, acquainting students with the essential role of FM in the delivery of healthcare systems.

## Goals and objectives

During the initial stages of developing the FM curriculum, core competencies were attained by considering international contexts and adapting to the local context. A preliminary draft of the curriculum (learning outcomes, teaching, or learning methods, and assessments) was developed. International and local FM educators were consulted for further refinement. A meeting was conducted with senior FM consultants, specialists, and senior general practitioners (GPs) to determine the competencies and attributes essential for general practice in the region. There was a consensus on the core knowledge, skills, and attitudes that guided teaching, learning, and assessment, which were further revised and refined.

During the subsequent academic years, the FM curriculum was revised based on the curricula of the FM program provided by the Society of Teachers in Family Medicine [20]. The curriculum included a list of standard and essential presentations which medical students would experience during their FM training. Additionally, for the application of the FM program, the CoM adopted the WONCA checklist which aimed to ensure that students grasp the essential competencies for understanding and learning FM in their regional context [21]. **Appendix 1** outlines the learning outcomes and teaching and assessment methods of our FM program.

## Educational strategies

The core content of the FM program focused on areas relevant to general practice, emphasising the biopsychosocial model of patient care and patient-centredness. These elements included health promotion, prevention, child and women’s health, and chronic health conditions such as diabetes mellitus, hypertension, bronchial asthma, and common undifferentiated conditions. Moreover, ethical dilemmas, patient safety, and evidence-based healthcare principles were emphasised, including the critical appraisal of articles, as specific skills that students were expected to practice.

**Fig. 1:**
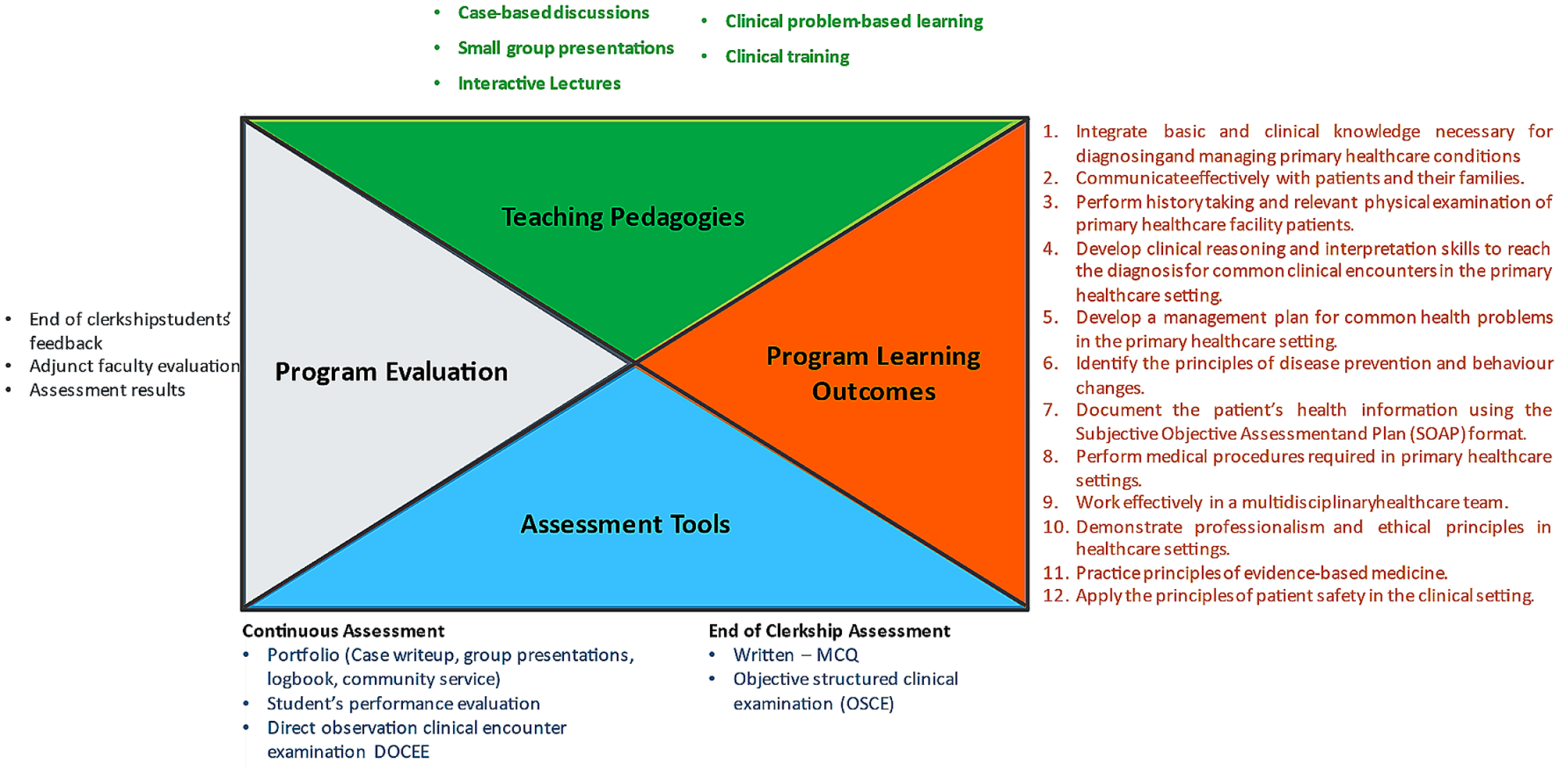
A snapshot of the family medicine program with learning outcomes, teaching pedagogies, assessment tools, and evaluation

Along with experiential learning at training sites, diverse teaching and learning methods were employed, including small-group discussions, interactive lectures, and clinical problem-based learning [22]. Figure 1 illustrates a snapshot of the FM program learning outcomes, teaching pedagogies, assessment tools, and evaluation.

## Implementation

The clinical training of the FM program was conducted in the affiliated public and private primary healthcare centres (PHCCs). The students were divided into small groups of 5–6 each and they were rotated between two PHCCs, three weeks each, for convenience and diversity. The FM department did not have a full-time clinical faculty with a practising license which posed challenges of recruiting dedicated ACF at PHCCs.

Although there was official memorandums of understanding with the affiliated public and private PHCCs, ACF had limited time for dedicated teaching, and some physicians were reluctant to accept students in their clinics because of their demanding clinical responsibilities. To overcome this hurdle, the CoM offered a part-time contract with academic titles to encourage the primary healthcare physicians as ACF for their dedicated clinical training.

Moreover, open communication channels were created among ACF through emails and regular meetings for direct discussion of their concerns. Later, ACF of PHCCs were paid an honorarium by the UoS according to their qualifications, teaching hours, and for their roles as coordinators. At each training site, one ACF was nominated as a clinical coordinator to facilitate teaching and assessment and to communicate students’ progress to the FM department.

To mitigate inconsistency in teaching and assessment, we organised several faculty development workshops during the initial implementation of the FM program. The primary purpose of workshops was to familiarise clinicians with essential components of experiential learning in the clinical environment. The workshop topics included teaching and learning in ambulatory care setting, giving feedback, assessment of clinical competence, and portfolio assessment. These workshops were repeated on different occasions to fit clinicians’ busy schedules. Transfer of training from workshops to training sites was further supported by regular visits of the CoM faculty to the training sites and by conducting formative clinical examinations, which provided an opportunity for feedback to the students.

Assessment is an essential driver of learning, and we used a wide spectrum of assessment tools including Direct Observation Clinical Encounter Examination (DOCEE) [23], Objective Structured Clinical Examinations (OSCE) [24], students’ performance during clinical training (professional behaviour and interaction), written exams, and portfolios. Students were expected to submit case write-ups written in a structured format with reflective reports and presentations related to incidents that raised ethical concerns about patient safety, medical error or near miss. Furthermore, students submitted critical appraisals on diagnostic and therapeutic published articles. Finally, the students were instructed to submit a logbook with a record of the clinical activities in which they were involved during their clinical training such as clinical encounters, procedures, and tutorials.

## Evaluation and feedback

Continuous improvement was a key focus during the process of the development, implementation, and evaluation of the FM program. We regularly assessed and evaluated the curriculum’s effectiveness, making necessary adjustments to enhance its impact. The primary purpose of the evaluation was to collect information about the gaps in learning and to identify different sections of the program. The FM department utilised various sources of information to evaluate clerkships including students’ end- of clerkship feedback as well as ACF feedback to reflect on and to share their experiences, discuss challenges, and offer solutions. The students’ surveys at the end of the clerkship were distributed to explore their experiences about the training sites, number and variety of clinical encounters, performance feedback, and their overall satisfaction with the training at each PHCC. All students’ feedback was then sent to ACFs in all involved PHCCs at the end of each rotation to further explore and discuss the challenges and barriers to the delivery of the FM program. Finally, the evaluation reports were reviewed during the annual department meetings where clinical coordinators from training sites were invited to enrich discussions and to provide objective feedback.

**Fig. 2:**
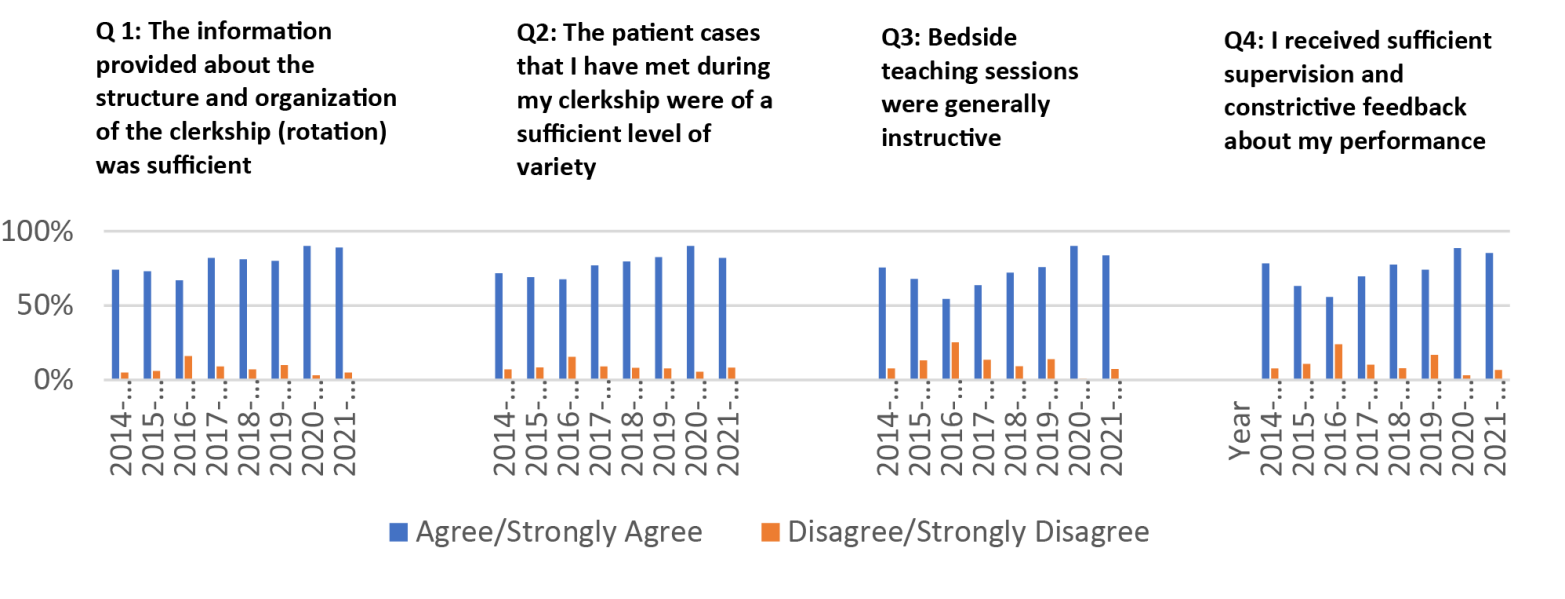
Students’ responses to statements 1–4 of the survey for the evaluation of the FM program

**Fig. 3:**
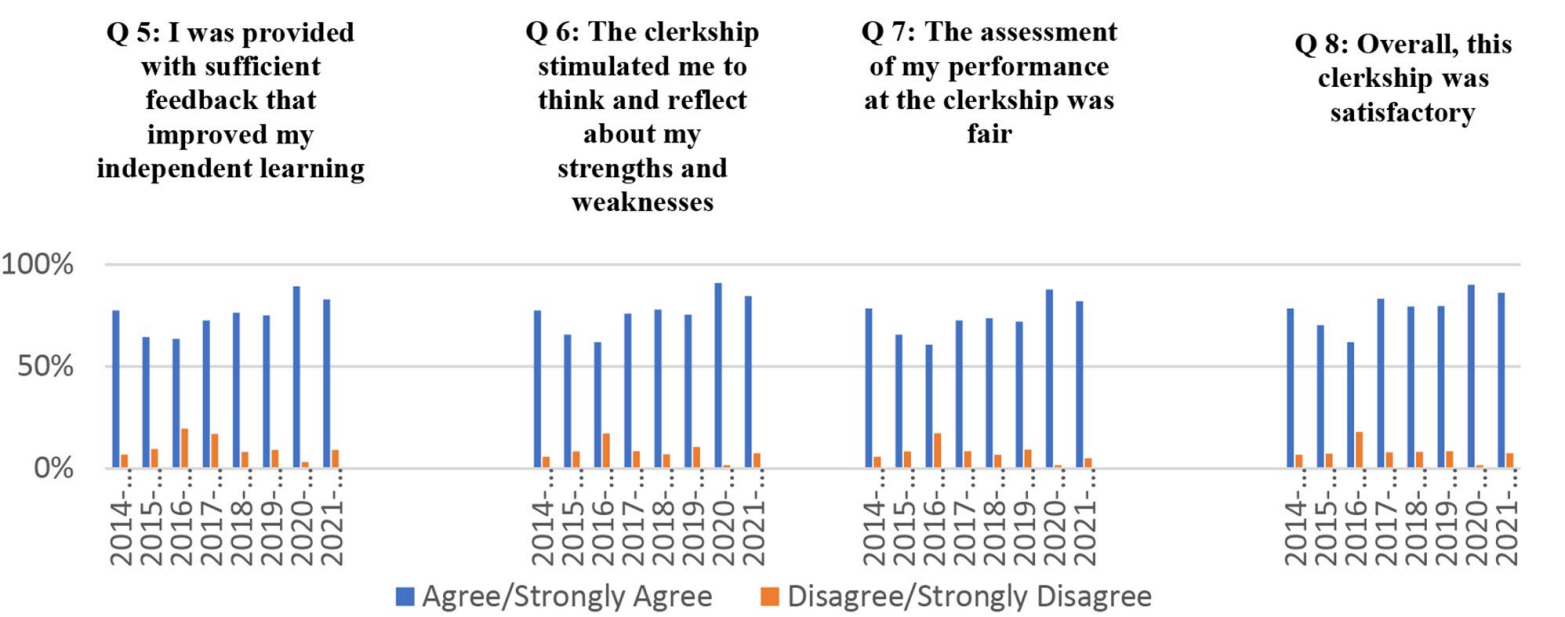
Students’ responses to statements 5–8 of the survey for the evaluation of the FM program

The student’ responses to the survey questions were analysed using spread excel sheet to generate bar charts representing percentages for each of the questions (Figs. 2 and 3). Students’ responses to the open-ended questions were reviewed and summarised for discussions and decision-making.

## Results

Over the course of eight academic years, 804 out of 1020 (79%), 49 ACF and three CoM faculty participated in the development, implementation, and evaluation of the FM program. The survey results showed that students had overall positive experiences during their rotations at most of the clinical sites they visited. Majority of students agreed/ strongly agreed with the adequacy of the information provided, the variety of clinical cases, the quality of bedside teaching, and supervision. The descriptive analysis of the students’ responses to statements 1–4 are displayed in Fig. 2.

Similarly, the students agreed/ strongly agreed with the educational quality of the feedback and the assessment methods. Moreover, most students agreed that the FM program stimulated them to reflect on their academic performance. They also agreed/strongly agreed that the FM program was satisfactory, and their satisfaction was highest during the latter two years 2020 and 2021 (Fig. 3). The descriptive analysis of the students’ responses to statements 4–8 are shown in Fig. 3.

Students’ comments from the free-text options of the questionnaire revealed the following four aspects: (1) a variety of cases (2) skills gained, (3) helpful doctors, and (4) organised program. Students reported that many of their clinical encounters allowed them to gain experience about a broad range of medical conditions and commented that ‘there was a great variety of cases’ (2019–2020) which enabled them to ‘apply everything I was learning, and I got to see a wide variety of cases’ (2020–2021). Students also reported considerable skills gain during their clerkship, including hands-on patient care, focused history-taking, and communication skills. For instance, one student noted that: ‘This rotation helped me improve my communication skill in dealing with patients’ (2015–2016). Another student noted that: ‘We got the chance to take history and perform physical examinations to the patient and get feedback from the doctors’. (2019–2020). Students viewed the clerkship programme as well organised and that accessing their needed resources was easy.

‘It was very organised. Every student knew where they were assigned each day, whether general clinic, antenatal clinic, vaccination clinic, screening clinic, pharmacy, lab, or vitals. The doctors ensured that we benefited from every minute in the clinic”, (2016–2017).

Finally, students also commented that their preceptors helped teach them the skills necessary to succeed as physicians. They also said the clinical instructors were knowledgeable and always willing to answer questions and provide feedback to improve their skills: ‘The doctors were open to any question medical-wise and gave us multiple opportunities to take history and examine patients on our own, then report to them the findings’. (2016–2017). Another student noted: ‘… Health centres are very nice places to learn more about FM. Doctors are nice, have knowledge and experience, and are willing to teach and help’ (2018–2019).

In relation to improvement students commented on having dedicated faculty for clerkships, the need for faculty development for the clinical teachers, and the need for improvement at the teaching sites. They pointed out the need to have faculty members engaged in clerkships dedicated explicitly to the clinical training of medical students for better supervision: ‘Students should be supervised; it would be great if the doctors could listen to the histories since psychiatric history taking is very different from the usual history, and we need feedback’ (2021–2022). Similarly, another student noted that: ‘We need more engagement for history taking and more teaching by doctors’ (include year). Students also suggested the need for faculty development in teaching and student assessment to ensure future success: ‘We need more engagement for history taking and more teaching by doctors’ (2021–2022). Lastly, some students also commented on the need for dedicated clinical sites for the delivery of the FM program: ‘We need more facilities for this hospital for adequate training’ (include year).

Similarly, the responses by ACF in terms of strengths and areas for improvement of the FM program as summarised in Table 1.

**Table 1:** A summary of the evaluation of the family medicine program by adjunct clinical faculty

Strengths of the family medicine program	Suggestions for improvement
• The e-clinic (telemedicine) component provided a good opportunity to students to interact with patients. • The systematic rotations of the students among different clinics provided a wide variety of experiences. • The structure, learning objectives, teaching modalities, as well as the method of continuous quality improvement of the program helped students to acquire essential knowledge and competence. • Annual faculty meetings with the academic coordinators provided opportunity to discuss strengths and limitations of the program.	• Organize student training about the application of telemedicine. • Allocate appropriate number of students in each clinic to maximize benefit. • Engage interns and residents in clinical training of students, for effective peer-assisted learning. • Develop and disseminate a clear guideline for students’ attendance and compensation for absenteeism. • Organize regular case-based discussions, to enhance clinical reasoning and management skills. • Train adjunct clinical faculty to foster their teaching, and assessment skills in ambulatory care.

## Discussion

This work describes a systematic process of the development, implementation, and evaluation of the FM program at the CoM of UoS. Our work contributes to the scare literature on FM programs in EMR by highlighting the development, implementation, and evaluation of a community-based FM program in the UAE. The findings of our evaluation conducted over several years indicated that our students had overall positive experiences during their rotations at most of the clinical sites they visited. The majority of students agreed/ strongly agreed with the adequacy of the information provided, the variety of clinical cases, the quality of bedside teaching, and supervision, as well as educational quality of the feedback and the assessment methods. They also agreed/strongly agreed that the FM program was satisfactory, and their satisfaction was highest during the latter two years 2020 and 2021.

The FM program is a critical component of medical education, as it helps students learn how to apply their basic clinical skills and cognitive knowledge. At the initial stage of the curriculum development and implementation, it was necessary to consult both local and international experts in the FM field, which helped us to identify existing lacunas in the curriculum and the mechanisms to rectify the curricular defects. Drowos et al., have eluded that community-based faculty plays a major role in the clinical training of medical students, but getting their interest, attention, and protected time for training remain elusive [25]. The authors have argued that by providing payment to community preceptors can potentially enhance effectiveness of clinical training. Our FM department adopted a similar approach by offering academic titles and honorarium to the ACF.

The development and implementation of our FM program is appropriate in the local and regional context. This requires considering several aspects, including international and local consultations, students’ stage of expertise, duration of the clerkship, facilities available in the training sites, and preceptors’ teaching skills. The students’ stage of expertise significantly influences the design and core content of the FM program [26]. The FM program must be planned to allow students to gain the necessary core FM competencies, make meaningful links to previously learned content throughout the pre-clerkship and clerkship phases of the MBBS curriculum, and revisit that information and reflect on their learning over time [23]. In our study, a rigorous overview of the program contents, delivery and assessment was carried out by the FM department to ensure a consistent and uniform delivery of the curriculum.

Based on our experiences of running the FM program over the past decade, we propose some recommendations for the medical educators who are planning a to develop a similar curriculum. A well-coordinated alignment of the curriculum learning outcomes with teaching pedagogies and assessment is crucial [27]. It is fundamental to balance learning outcomes with the duration allotted for the clerkship. In our program, suggestions were made to improve students’ workload which could potentially enhance the program effectiveness. Similarly, its essential to incorporate the teaching and learning principles and core values of FM when developing and promoting an undergraduate curriculum as a career choice for medical students [28].

Institutional support is necessary for the successful implementation of the clerkship particularly in the presence of multiple training sites. Such support includes provision of transport, ACF honorarium based on their teaching efforts and student’s assessment and academic titles. In addition, there is a great need to obtain strong support from the administrators of public and private healthcare facilities to accommodate the training of medical students in healthcare facilities. The UoS administration has been supportive throughout the process of the development and implementation of the FM program.

Additionally, recruiting well trained and motivated faculty and ACF is a fundamental requirement for the successful implementation of the FM program. In our FM program, the clinical training and supervision in PHCCs were performed by preceptors who were full-time physicians working in the affiliated private and public clinics with a part-time adjunct faculty contract with the CoM. We ensured open communication channels between adjunct faculty physicians in affiliated clinics through email and regular meetings, clarifying any queries and encouraging more physicians to become part of the adjunct faculty.

Achieving consistency and quality control in teaching and evaluation is critical when implementing a FM program particularly by improving teaching styles of the clinical faculty [29]. To mitigate inconsistency in teaching and assessment, we organised several faculty developments workshops during the initial implementation of the FM program. Continuous monitoring and evaluation of each training site are needed to ensure uniformity in the teaching and assessment processes [30, 31]. Lastly, it is imperative to facilitate variations in clinical exposure. Our FM program was structured to allow students to rotate between two PHCCs, spending three weeks at each centre to enhance students’ clinical exposure to a variety of cases. Within each PHCC, students rotated between different ACF in various service clinics such as antenatal, well-baby, non-communicable disease, periodic check, and general clinics. Furthermore, the students had diverse teaching experiences with different physicians. Based on students’ feedback, some sites were excluded from training because of the limited patient encounters or the unavailability of ACF to supervise clinical training.

In contemplating future directions for the advancement of our FM program, the department is considering several key initiatives that could significantly enhance the delivery of the curriculum. Foremost among these is to extend the duration of the program. Providing an extended and immersive experience is essential for deeper understanding of the breadth and depth of primary care, fostering a genuine appreciation for the specialty, and consequently, positively influencing their career choices [28, 32].

Another crucial perspective for the seamless progression of the program involves the integration of a new telemedicine curriculum. Incorporating a dedicated course on telemedicine is viewed to equip learners with essential skills for effective virtual patient care [33]. Furthermore, it aligns with the evolving landscape of healthcare delivery. This forward-looking approach ensures that medical students utilize technological advancements to deliver patient-centered care, preparing them for the dynamic challenges of modern medical practice.

Additionally, another strategic initiative under consideration is the development of a comprehensive training program on electronic medical records (EMRs). Recognizing the increasing reliance on EMRs in healthcare, the FM department aims to prepare students with the proficiency to navigate and utilize these systems effectively. Such a training program is perceived as imperative for providing high-quality care to patients and communities, empowering future physicians to seamlessly integrate electronic record-keeping into their practice and contributing to improved patient outcomes and overall healthcare service efficiency [34].

Other plans for enhancing the effectiveness of the FM program involve incorporating senior FM residents into the teaching process to address the shortage of clinical supervisors and accommodate the growing number of medical students. Evidence suggests that residents play a significant role as primary instructors for medical students, especially in practical clinical skills [35–37]. Based on the feedback, the FM department is planning to include a quality improvement course by engaging undergraduate medical students in the audit process not only to enhance their comprehension of the subject but also to contribute to nurturing future consultants with the essential skills for critical appraisal, leadership [38], Based on the feedback, the FM department is planning to include a quality improvement course by engaging undergraduate medical students in the audit process not only to enhance their comprehension of the subject but also to contribute to nurturing future consultants with the essential skills for critical appraisal, leadership [39, 40].

## Limitation

Our work did not include all stakeholders required in program evaluation including administrators, organisers, and coordinators. Our research primarily reported the findings of quantitative data with limited qualitative analysis, which might be a limitation or shortcoming of our study.

## Conclusion

In this paper, we have reported a successful implementation and evaluation of our FM program with a positive and impactful evaluation by medical students and ACF. However, for future development of such programs, more attention should be paid for suitable representation of FM program in the MBBS curriculum with focused, individualised, and personalised educational plans for medical students.

### Electronic supplementary material

Below is the link to the electronic supplementary material.

Supplementary Material 1

## Data Availability

The datasets used and/or analysed during the current study are available from the corresponding author, NS, if requested.
