# Supplementary material for: Development, implementation, and evaluation of an undergraduate family medicine program in the United Arab Emirates
Source: BMC Med Educ. 2024 Mar 20;24:311. doi: 10.1186/s12909-024-05134-6 (PMC10953071; doi:10.1186/s12909-024-05134-6)
Supplement: Supplementary file 1 — Supplementary Material 1 [file 12909_2024_5134_MOESM1_ESM.docx]

**Appendix**

**Table: The learning outcomes, teaching, and assessment methods of the family medicine program.**

| **Clerkship learning outcomes** | **Teaching and learning strategies** | **Assessment tools** |
| --- | --- | --- |
| 1. Integrate basic and clinical knowledge necessary for diagnosing and managing primary healthcare conditions. | - Experiential learning in a clinical and simulated setting - Small group discussions - Lectures - Clinical PBL - Students’ presentations | - DOCEE - OSCE - Clerkship Student’s Performance Evaluation - MCQs - Student’s presentation |
| 1. Communicate effectively with patients and their families. | - Experiential learning in a clinical and simulated setting | - DOCEE - OSCE - Clerkship Student’s Performance Evaluation |
| 1. Perform history taking and relevant physical examination of primary healthcare facility patients. | - Experiential learning in a clinical and simulated setting | - DOCEE - OSCE - Clerkship Student’s Performance Evaluation |
| 1. Develop clinical reasoning and interpretation skills to reach the diagnosis for common clinical encounters in the primary healthcare setting. | - Experiential learning in a clinical and simulated setting - Small group discussions - Clinical PBL | - DOCEE - OSCE - Clerkship Student’s Performance Evaluation - MCQs |
| 1. Develop a management plan for common health problems in the primary healthcare setting. | - Experiential learning in a clinical and simulated setting - Small group discussions - Lectures - Clinical PBL - Students’ presentations | - DOCEE - OSCE - Clerkship Student’s Performance Evaluation - MCQs |
| 1. Identify the principles of disease prevention and behaviour changes. | - Experiential learning in a clinical and simulated setting - Small group discussions - Lectures - Clinical PBL | - DOCEE - OSCE - MCQs |
| 1. Document the patient’s health information using the Subjective Objective Assessment and Plan (SOAP) format. | - Experiential learning in a clinical and simulated setting - Small group discussions - Lectures | - Portfolio |
| 1. Perform medical procedures required in primary healthcare settings. | - Experiential learning in a clinical and simulated setting | - DOCEE - OSCE |
| 1. Work effectively in a multidisciplinary healthcare team. | - Experiential learning in a clinical and simulated setting - Small group discussions | - Clerkship Student’s Performance Evaluation |
| 1. Demonstrate professionalism and ethical principles in healthcare settings. | - Experiential learning in a clinical and simulated setting - Small group discussions - Lectures - Clinical PBL - Students’ presentations | - DOCEE - OSCE - Clerkship Student’s Performance Evaluation - MCQs - Portfolio |
| 1. Practice principles of evidence-based medicine. | - Experiential learning in a clinical and simulated setting - Small group discussions - Lectures - Clinical PBL - Students’ presentations | - DOCEE - OSCE - Clerkship Student’s Performance Evaluation - MCQs - Portfolio - Student’s presentation |
| 1. Apply the principles of patient safety in the clinical setting. | - Experiential learning in a clinical and simulated setting - Small group discussions - Lectures - Clinical PBL | - DOCEE - OSCE - Clerkship Student’s Performance Evaluation - MCQs - Student’s presentation |
